# Multilevel analysis of individual and community level factors associated with the application of cow dung and oil on the umbilical cord stump in Ethiopia

**DOI:** 10.3389/fped.2022.1044056

**Published:** 2022-11-07

**Authors:** Anteneh Mengist Dessie, Habtamu Geremew, Sefineh Fenta Feleke, Denekew Tenaw Anley, Kalayu Brhane Mruts, Chalachew Yenew, Berihun Bantie, Natnael Moges Misganaw, Gashaw Kerebeh, Asaye Alamneh Gebeyehu, Desalegn Tesfa Asnakew, Rahel Mulatie Anteneh

**Affiliations:** ^1^ Department of Public Health, College of Health Science, Debre Tabor University, Debre Tabor, Ethiopia; ^2^ College of Health Science, Oda Bultum University, Chiro, Ethiopia; ^3^ Department of Public Health, College of Health Science, Woldia University, Woldia, Ethiopia; ^4^ School of Public Health, College of Medicine and Health Sciences, Debre Berhan University, Debre Berhan, Ethiopia; ^5^ Department of Adult Health Nursing, College of Medicine and Health Sciences, Debre Tabor University, Debre Tabor, Ethiopia; ^6^ Department of Pediatrics and Child Health Nursing, College of Health Science, Debre Tabor University, Debre Tabor, Ethiopia

**Keywords:** cow dung, Ethiopia, multilevel modeling, oil, umbilical cord stump

## Abstract

**Background:**

Hygienic umbilical cord care is one of the essential interventions advocated to reduce neonatal mortality. However, traditional cord care measures-applying cow dung and oil-that have harmful health consequences are commonly practiced in Ethiopia. Hence, in this study, it was planned to analyze individual and community-level factors associated with the application of cow dung and oil on the umbilical cord stump in Ethiopia.

**Methods:**

Data from the 2016 Ethiopian demographic and health survey were used to identify individual and community level factors associated with women's practice of applying cow dung and oil on the umbilical cord stump of their neonate. Taking into account for the hierarchical structure of the data; multilevel binary logistic regression analysis has been employed to a nationally representative weighted sample of 7,168 women.

**Results:**

In Ethiopia, 780 (10.88%) with 95% CI (10.18–11.62) women apply oil and/or cow dung on the neonate's umbilical cord stump. Age increase by one year [AOR = 0.97; 95% CI (0.94–0.99)] and giving birth in a health facility [AOR = 0.61; 95% CI (0.42–0.89)] were individual-level factors that reduced women's practice of applying cow dung and oil on the umbilical cord stump of their neonate. Whereas, rural residence [AOR = 2.54; 95% CI (1.28–5.06)] was the predictor at the community level that raised the practice of applying cow dung and oil on the neonate's umbilical cord stump.

**Conclusion:**

This nationwide study revealed that a significant number of mothers in Ethiopia still apply cow dung and/or oil on the umbilical cord stump of their neonates. Both the individual and community level characteristics: maternal age, place of delivery, and residence were found to have significant inﬂuence on the practice of applying cow dung and/or oil on the umbilical cord stump in Ethiopia. Thus, to reduce neonatal mortality due to avoidable umbilical cord infections, clean cord care practice strategies should be designed by considering these factors.

## Introduction

Globally, an estimated 2.4 million neonatal deaths were reported in 2019, the vast majority of which occurred in low and middle-income countries (1). Neonatal mortality persisted to be an important public health concern in Ethiopia, exhibiting retarded reduction over years (1, 2). With a higher rate of unclean home deliveries, neonatal sepsis and neonatal tetanus contribute a big share to the observed high neonatal mortality; particularly in underdeveloped settings like Ethiopia (3–6).

Hygienic umbilical cord care is one of the essential interventions advocated to reduce neonatal mortality (1, 7). Proper cord care with the appropriate antiseptic solution is recommended to prevent infection and promote healing of the umbilical stump, particularly in resource-constrained settings where the environment is not clean and conducive (8, 9). However, traditional cord-care measures that have harmful health consequences are commonly practiced in Ethiopia; among this application of cow dung and oil on the umbilical cord stump are the major public health threats (10–12). Accordingly, only 46.1% of newborns in Ethiopia were found to receive cord care with recommended antiseptic solutions (13).

The umbilical stump is a common point of entry for microorganisms in neonates (14). This is further worsened by the application of unsafe external substances like cow dung and oil (14, 15). Cow dung is a special place where spores of *Clostridium tetani* and other important microorganisms live (15). Hence applying this substance on the umbilical stump of neonates would mean inoculating those microorganisms into the newborns' circulation (7). Reports also indicated a significantly higher risk of umbilical infection associated with the application of oil on the umbilical stump (16).

Identifying factors associated with such malpractices is essential for improving neonatal survival through evidence-based care. In Ethiopia however, few studies investigated cord care practice; yet they used a small sample size representing a specified locality and none of the studies have tried to look at individual and community levels factors simultaneously. Additionally, using a standard binary regression model to regress variables at various levels will result in bias and power loss. Thus, this study aimed to simultaneously analyze the individual and community level factors associated with the application of cow dung and/or oil on the umbilical cord stump in Ethiopia with the application of multilevel modeling and utilizing nationally representative data.

## Methods

### Study design, area and period

This was a population-based cross-sectional study conducted from January 18 to June 27, 2016 in Ethiopia which is located in the Eastern tip of Africa. Ethiopia is the second-most populous nation in Africa, with over 100 million populations (CSA, 2012). Administratively, it is divided into nine regions and two city administrations subdivided into 68 zones, 817 districts, and 16,253 kebeles (lowest administrative unit in the country).

### Data sources, study populations and sampling

This cross-sectional study analyzed the 2016 Ethiopia Demographic Health Survey (EDHS) existing data. Approval letter for the use of this data was gained from the Measure DHS and the data set was downloaded from the Measure DHS website www.meauredhs.com. The survey used a two-stage stratified cluster sampling design to select eligible participants. In the first stage, 645 (202 from urban and 443 from rural areas) clusters/Enumeration Areas (EAs) were randomly selected from the lists of EAs developed from the 2007 population and housing census after stratification of the 11 regional states into urban and rural areas. Second, a fixed number of 28 households per cluster/EA/were selected using systematic random sampling. Consequently, a total of 18,008 households were selected. Finally, only 15,683 households contained eligible women in reproductive groups who responded completely.

A total of 7,193 women were interviewed to provide information umbilical cord care of their under-five child, and 10,641 under-five children's details were gathered. But in this study, in order to obtain recent information and reduce recall bias, only the most recent live births (7,193 children) were taken into account. In the end, a study analyzed a total of 6,775 (weighted 7,168) mothers of under-five children after dropping an observation those who don't know whether they apply anything on the umbilical cord stump or not (418 observations). Weighting factors were used in sampling to make samples match the population.

### Variables of the study

*Dependent variable:* The outcome variable in this study was whether cow dung and/or oil were applied on the umbilical cord stump of the recent live birth or not. It is a binary variable categorized as “Yes” or “No”.

*Explanatory Variables:* They broadly categorized as individual and community-level factors. Age of women, birth order, marital status, religion, education of women and husbands, working status, household wealth index, distance to health facility, media exposure, ANC visit, place of delivery, and PNC visit are all factors that are considered at the individual level. Community level factors include region, place of residence, distance to health facility, community poverty, community women's education, and community media exposure.

The aggregated community level factors were constructed by aggregating individual-level characteristics at the community (cluster) level. Categorization of the aggregate variables was done as high or low based on the median value because they were not normally distributed. Community poverty was defined as the proportion of households in the poorest and poorer quintile. Community women's educational level was defined as the proportion of women with a minimum of primary level of education. Community media exposure was defined as the proportion of women exposed to at least one type of media, such as radio, newspaper or television for at least once a week. Distance to health facility was defined as the proportion of households with big problem of distance to health facility (big problem and not a big problem).

### Data processing and analysis

The data cleaning and all statistical analyses were performed using Stata V.16.0. The analysis was conducted after sample weights were applied to compensate for the unequal probability of selection between the strata that were geographically defined, as well as for non-responses. Descriptive statistics using frequency and percentage were used to get an overview of the selected variables.

A two-level multilevel binary logistic analysis was employed in order to account for the hierarchical structure of the DHS data i.e., individuals (level 1) were nested within communities (level 2). This analysis is important to estimate both independent (fixed) effects of the explanatory variables and community-level random effects on the application of cow dung and oil on the umbilical cord stump. The model is specified as follow:

$$\mathrm{Logit}\;\left(P_{\mathrm{ij}}\right)\;=\;\beta_{0}\;+\;\beta_{1}X_{\mathrm{ij}}\;+\;\beta_{2}Y_{\mathrm{ij}}\;+\;u_{j}\;+\;e_{\mathrm{ij}}$$

Where, P_ij_ is the probability of cow dung and/or oil application on the umbilical cord stump for the ith child in the jth community; the *β*'s indicates the fixed coefficients; X and Y refer to individual and community-level variables respectively, u_j_ showed the random effect (effect of the community on cow dung and/or oil application on the umbilical cord stump) for the jth community and e_ij_ showed random errors at the individual levels.

First, bi-variable multilevel binary logistic regression models were fitted and all variables with a *p-*value <0.25 at bi-variable analysis were selected to build the 3 models (model I–III). Four models were fitted. A null model which was the unconditional model included the outcome variable only to estimate the random intercept at cluster level and the variation in the odds of cow dung and/or oil application on the umbilical cord stump between communities. Model I included outcome and individual-level variables, model II included outcome and community-level variables, and model III included the outcome variables, and both individual- and community- level variables. Then, the model that had the highest log likelihood or lowest deviance and Akaike Information Criterion (AIC) was chosen as the best model and used to estimate the association between independent factors and the outcome variable. The significance of the difference between the models was also evaluated using the chi square likelihood-ratio test. Finally, the fixed effects for the multivariable multilevel binary logistic regression model were reported as adjusted odds ratios (AOR) with 95% confidence intervals (CI), and statistical significance was declared at *p* value <0.05.

In addition, measures of variation (random effects) were assessed using several indicators such as area variance with 95% CI, the intra-class correlation coefficient (ICC), proportional change in variance (PCV), and the median odds ratio (MOR). The presence of multicollinearity was checked among independent variables using standard error at the cutoff point of ±2 and there was no multicollinearity.

## Results

### Socio-demographic characteristics of respondents

In this study, a total weighted sample of 7,168 mothers of under-five children was included. The median age of mothers was 29 years old with Inter Quartile Rang (IQR) of 25 to 35 years. Most of them were in the age group 25–34 years 3,587 (50.04%), Orthodox 2,650 (36.96%), married/in union 6,712 (93.63%), and were not exposed to media 5,843 (81.51%). Regarding educational status, nearly two-third 4,600 (64.18%) of women and nearly half of their husbands 3,203 (47.73%) had no formal education (Table 1).

**Table 1:** Weighted socio-demographic and economic characteristics of respondents EDHS 2016 (*N* = 7,168).

Variables	Frequency	Percentage
Mother's age (years)		
15–24	1,700	23.72
25–34	3,587	50.04
≥35	1,881	26.24
Place of residence		
Urban	811	11.31
Rural	6,357	88.69
Marital status		
Married/in union	6,712	93.63
Not married/in union	456	6.37
Religion		
Orthodox	2,650	36.96
Muslim	2,698	37.63
Protestant	1,590	22.19
Others	230	3.21
Woman's educational status		
No education	4,600	64.18
Primary	2,020	28.18
Secondary	364	5.07
Higher	184	2.57
Educational status of husband		
No education	3,203	47.73
Primary	2,600	38.74
Secondary	556	8.29
Higher	321	4.77
Don’t know	32	0.47
Working status of mother		
Working	2,022	28.21
Not working	5,146	71.79
Household wealth quintile		
Poor	3,216	44.86
Middle	1,517	21.17
Rich	2,435	33.97
Media exposure		
Has media exposure	1,325	18.49
Has no media exposure	5,843	81.51
Distance to health facility		
Big problem	4,240	59.16
Not big problem	2,928	40.84

### Reproductive history and child characteristics

Regarding maternal health service utilization, 4,408 (61.50%) of women had at least one ANC visit, whereas only 1,123 (15.67%) had PNC visits. Furthermore, 2,866 (39.98%) of the mothers had five and above children ever born. Three thousand seven hundred thirty (52.03%) of the total recent children included in this study were males, and 2,994 (41.77%) belonged to the birth order of two to four (Table 2).

**Table 2:** Reproductive history and their recent child characteristics of women who gave birth within the last five years preceding the survey in Ethiopia, EDHS 2016 (*N* = 7,168).

Variables	Frequency	Percentage
ANC visit		
Yes	4,408	61.50
No	2,760	38.50
Place of delivery		
Home	5,124	71.48
Health institution	2,044	28.52
PNC visit		
Yes	1,123	15.67
No	6,045	84.33
Inter-birth interval		
Not applicable	1,319	18.40
<33 months	2,354	32.84
≥33 months	3,495	48.76
Birth order		
1	1,308	18.25
2–4	2,994	41.77
≥5	2,866	39.98
Child sex		
Male	3,730	52.03
Female	3,438	47.97

### Community level variables

Four thousand seven hundred twelve (64.75%) of women were in the community where there is a big problem of distance to the health facility and 4,204 (58.66%) were from the community with high level poverty. Of all, 6,053 (84.44%) of respondents were from Amhara, Oromia, and SNNP (Table 3).

**Table 3:** Distribution of both actually collected and aggregated community level variables of the study, EDHS (2016) (*N* = 7,168).

Variables	Frequency	Percentage
Place of residence		
Urban	811	11.31
Rural	6,357	88.69
Region		
Tigray	489	6.82
Afar	66	0.93
Amhara	1,506	21.01
Oromia	3,012	42.01
Somali	264	3.68
Benishangul	80	1.11
SNNPR	1,535	21.42
Gambela	20	0.28
Harari	16	0.23
Addis Ababa	150	2.09
Dire Dawa	31	0.43
Community-level poverty		
Low	2,964	41.34
High	4,204	58.66
Community women education		
High	2,730	38.08
Low	4,438	61.92
Community media exposure		
High	2,769	38.63
Low	4,399	61.37
Distance to health facility		
Big problem	4,714	65.76
Not big problem	2,454	34.24

### Practice of cow dung and/or oil application on the umbilical cord stump

In Ethiopia, 769 (10.73%) and 13 (0.18%) of women apply oil and cow dung on the umbilical cord stump of their child respectively. Over all, the prevalence of cow dung and/or oil application on umbilical cord stump in Ethiopia was 780 (10.88%) with 95% CI (10.18–11.62). out of these mothers who apply cow dung and/or oil on umbilical cord stump of their child, 691 (88.6%) were from rural residents.

### Multilevel logistic regression analysis

The fixed effects (measure of association) and the random intercepts for the application of cow dung and/or oil on the umbilical cord stump are presented in Table 4. The null model therefore justified the use of multilevel modeling by showing that about 39.3% of the total variance in applying cow dung and/or oil on the umbilical cord stump was attributed to differences between communities (ICC = 0.393). About 5.6% of variation in applying cow dung and/or oil on the umbilical cord stump across communities was explained in the full model, as indicated by the PCV. Factors associated with the umbilical cord stump application of cow dung and/or oil was identified using Model III, which had the lowest deviance (4,368.615) and AIC (4394.615). All interpretations and conclusions drawn from the data were therefore based on model III. The MOR of 3.85 in the final model showed the effects of community heterogeneity, suggesting women from the community with the highest risk of applying cow dung and/or oil on the umbilical cord stump had 3.85 times higher odds of applying cow dung and/or oil as compared with those women from the community with the lowest risk.

**Table 4:** Multilevel binary logistic regression analysis for factors associated with umbilical cord stump application of cow dung and/or oil in Ethiopia, EDHS 2016.

Variables	Null model	Model I (AOR with 95% CI)	Model II (AOR with 95% CI)	Model III (AOR with 95% CI)
Age of the mother		0**.**97 **(**0**.**94,0**.**99)		0**.**97 **(**0**.**94,0**.**99)
Marital status				
Married/in union		1		1
Unmarried		1.17 (0.66,2.09)		1.18 (0.66,2.11)
Religion				
Orthodox		1		1
Muslim		0.76 (0.49,1.16)		0.77 (0.50,1.20)
Protestant		1.20 (0.76,1.89)		1.19 (0.74,1.90)
Others		1.57 (0.66,3.75)		1.57 (0.65,3.77)
ANC visit				
Yes		1.25 (0.82,1.89)		1.24 (0.82,1.89)
No		1		1
Place of delivery				
Home		1		1
Health institution		0**.**59 **(**0**.**41,0**.**84)		0**.**61 **(**0**.**42,0**.**89)
Media exposure				
Yes		1.14 (0.82,1.59)		1.20 (0.86,1.69)
No		1		1
Residence				
Urban			1	1
Rural			2**.**65 **(**1**.**38,5**.**10)	2**.**54 **(**1**.**28,5**.**06)
Community poverty				
Low			1	1
High			0**.**63 **(**0**.**41,0**.**97)	0.66 (0.42,1.05)
Distance to health facility				
Not big problem			1.18 (0.79,1.76)	1.24 (0.82,1.87)
Big problem			1	1
***Measure of variation***				
Area variance (95%CI)	2.13 (1.62,2.80)	1.95 (1.49,2.54)	2.03 (1.55,2.67)	2.01 (1.56,2.61)
ICC (%)	39.3	37.2	38.2	37.9
PCV (%)	Ref.	8.5	4.7	5.6
MOR	4.00	3.77	3.87	3.85
***Model comparison***				
Deviance (-2LL)	4,445.843	4,383.134	4,429.182	4,368.615
AIC	4,449.843	4,403.134	4,439.182	4,394.615

After adjusting the model with all the variables having *p*-value < 0.25 on the bi-variable multilevel binary logistic regression women's age, residence and place of delivery were significantly associated with umbilical cord stump application of cow dung and/or oil in Ethiopia. As age of women increases in one year the odds of applying cow dung and/or oil on the umbilical cord stump decreases by 3% [AOR = 0.97; 95% CI (0.94–0.99)]. The odds of applying cow dung and/or oil on the umbilical cord stump among rural women were 2.54 [AOR = 2.54; 95% CI (1.28–5.06)] higher as compared with urban women. The women who gave birth of the indexed child at health institution have 0.61 [AOR = 0.61; 95% CI (0.42–0.89)] times lower odds of applying cow dung and/or oil on the umbilical cord stump as compared with those who gave birth of the indexed child at home.

## Discussion

There were published standards for the care and management of neonates during their neonatal period in order to decrease neonatal mortality. Numerous published standards revolve around umbilical cord stump care because it is one of the significant factors influencing a newborn's risk of infection and mortality. When we observed the trend of nations with effective obstetric care and a low newborn mortality rate, one of the proposed courses of action was to support spontaneous umbilical cord drying, a cord care practice that is advised by the WHO (8). According to the study conducted in Pakistan, application of chemical in the umbilical cord stump can end with umbilical cord infection and be a factor for infant mortality (17, 18).

Thus, this study attempted to assess the individual and community-level determinants of umbilical cord stump practice in Ethiopia based on the EDHS 2016. According to this study, 769 (10.73%) and 13 (0.18%) women apply oil and cow dung on the umbilical cord stump of their child, respectively. Overall, the prevalence of cow dung and/or oil application on umbilical cord stumps in Ethiopia was 780 (10.88%) with 95% CI (10.18–11.62). This finding is lower than the studies from Mizan-Tepi University Teaching Hospital Southwest Ethiopia (36.6%) (10), Gulomekada District Eastern Tigray (60%) (19), Nigeria (38.6%) and (67.3%) (20, 21), and Rwanda (54%) (22). This might be attributed to the difference in methods used, study settings, and sample size enrolled. The variation might also be due to expanding health care coverage, increased awareness and information, and maternal health services now a day than before (23). In addition, in this study, we only took into account oil and cow dung, while other researchers took into account any substance applied to the umbilical cord stump (10, 20–22), which may explain the lower prevalence of this practice in this study. Activities to promote positive behavior change regarding umbilical cord care should be practiced to more lower the prevalence of improper cord care and its consequence.

Age of the mother, place of delivery, and residence were found to be important predictors in the multi-level analysis to evaluate individual and community-level factors that affect the application of cow dung and/or oil to the stump of the umbilical cord in Ethiopia. The likelihood of applying cow dung and/or oil on the stump of the umbilical cord stump drops by 3% as a woman's age rises over the course of a year [AOR = 0.97; 95% CI (0.94–0.99)]. A study from Pakistan (18) supported this. This might be linked with the increase in level of awareness as women become more aged and experienced. However, a controversial finding from Ethiopia's eastern Tigray region claimed that mother age is not a major factor for the practice of stumping the umbilical cord (19).

When compared to mothers who gave birth at home, those who had the indexing child at a health facility had a 39% [AOR = 0.61; 95% CI (0.42–0.89)] reduced the likelihood of putting oil and/or cow dung to the umbilical cord stump. This may be because women were given advice on important newborn care practices and approaches by medical personnel while they were hospitalized. The results highlight the need for further initiatives to boost the prevalence of institutional delivery and sanitary birthing procedures for deliveries taking place outside of healthcare facilities (24, 25). The odds of applying cow dung and/or oil on the umbilical cord stump among rural women were 2.54 [AOR = 2.54; 95% CI (1.28–5.06)] higher as compared with urban women. This is in line with the study done in Ethiopia (19), and Pakistan (18). A probable reason for this difference might be easy access to healthcare services and better education in urban areas as compared to rural areas (26).

In contrast to research from Nigeria (20) and Bangladesh (24), poor infant umbilical cord care (applying cow dung and/or oil) was not significantly influenced by media exposure, marital status, income, or ANC visits in this study. This could be due to the fact that Health Extension Workers (Professional Maternal and Child Health Care Providers at Home Level) visit all moms regardless of their educational position, money, or media exposure in Ethiopia (27). Additionally, there is a high rate of ANC follow-up visits, which contribute to better understanding and application of fundamental newborn care, including umbilical cord stump care (23).

## Conclusion

This nation-wide study on umbilical cord stump application of cow dung and/or oil in Ethiopia revealed that significant number of mothers still apply such things on the umbilical cord stump of their neonate. The risk of cord infection was likely to be raised by the application of oil and cow dung to the cord, which is against WHO recommendation. In this study both the individual and community level characteristics were found to have significant inﬂuence on the practice of applying cow dung and/or oil on the umbilical cord stump. Maternal age and place of delivery were the factors that inﬂuence umbilical cord stump application of cow dung and/or oil at the individual level. The communities in which the women reside (urban/rural) also play a significant role on women's practice of applying cow dung and/or oil on umbilical cord stump. Thus, to reduce neonatal mortality due to avoidable umbilical cord infections, health education on proper cord care practices should be integrated at the community level including rural residents and further initiatives should be there to boost the prevalence of institutional delivery and sanitary birthing procedures for deliveries taking place outside of healthcare facilities.

## Data Availability

The raw data supporting the conclusions of this article will be made available by the authors, without undue reservation.
